# Increase in the Number of Bone Marrow Osteoclast Precursors at Different Skeletal Sites, Particularly in Long Bone and Jaw Marrow in Mice Lacking IL-1RA

**DOI:** 10.3390/ijms21113774

**Published:** 2020-05-27

**Authors:** Giuliana Ascone, Yixuan Cao, Ineke D.C. Jansen, Irene Di Ceglie, Martijn H.J. van den Bosch, Arjen B. Blom, Peter L.E.M. van Lent, Vincent Everts, Teun J. de Vries

**Affiliations:** 1 Experimental Rheumatology, Radboud University Medical Center, P.O. Box 9101, 6500 HB Nijmegen, The Netherlands; Giuliana.Ascone@radboudumc.nl (G.A.); Irene.DiCeglie@radboudumc.nl (I.D.C.); Martijn.vandenBosch@radboudumc.nl (M.H.J.v.d.B.); Arjen.Blom@radboudumc.nl (A.B.B.); Peter.vanLent@radboudumc.nl (P.L.E.M.v.L.); 2 Department of Oral Cell Biology and Functional Anatomy, Academic Centre for Dentistry Amsterdam (ACTA), University of Amsterdam and Vrije Universiteit Amsterdam, Gustav Mahlerlaan 3004, 1081 LA Amsterdam, The Netherlands; Yixuan.Cao@ibms.pumc.edu.cn (Y.C.); V.Everts@acta.nl (V.E.); 3 Department of Periodontology, Academic Centre for Dentistry Amsterdam (ACTA), University of Amsterdam and Vrije Universiteit Amsterdam Gustav Mahlerlaan 2004, 1081 LA Amsterdam, The Netherlands; Ineke.Jansen@acta.nl

**Keywords:** monocyte, myeoloid blast, early blast, osteoclast precursor, osteoclast, IL-1 signaling, jaw, vertebrae, long bone, calvaria, osteoclast heterogeneity

## Abstract

Recently, it was shown that interleukin-1β (IL-1β) has diverse stimulatory effects on different murine long bone marrow osteoclast precursors (OCPs) in vitro. In this study, interleukin-1 receptor antagonist deficient (*Il1rn^−/−^*) and wild-type (WT) mice were compared to investigate the effects of enhanced IL-1 signaling on the composition of OCPs in long bone, calvaria, vertebra, and jaw. Bone marrow cells were isolated from these sites and the percentage of early blast (CD31^hi^ Ly-6C*^−^*), myeloid blast (CD31^+^ Ly-6C^+^), and monocyte (CD31*^−^* Ly-6C^hi^) OCPs was assessed by flow cytometry. At the time-point of cell isolation, *Il1rn^−/−^* mice showed no inflammation or bone destruction yet as determined by histology and microcomputed tomography. However, *Il1rn^−/−^* mice had an approximately two-fold higher percentage of OCPs in long bone and jaw marrow compared to WT. Conversely, vertebrae and calvaria marrow contained a similar composition of OCPs in both strains. Bone marrow cells were cultured with macrophage colony stimulating factor (M-CSF) and receptor of NfκB ligand (RANKL) on bone slices to assess osteoclastogenesis and on calcium phosphate-coated plates to analyze mineral dissolution. Deletion of *Il1rn* increased osteoclastogenesis from long bone, calvaria, and jaw marrows, and all *Il1rn^−/−^* cultures showed increased mineral dissolution compared to WT. However, osteoclast markers increased exclusively in *Il1rn^−/−^* osteoclasts from long bone and jaw. Collectively, these findings indicate that a lack of IL-1RA increases the numbers of OCPs in vivo, particularly in long bone and jaw, where rheumatoid arthritis and periodontitis develop. Thus, increased bone loss at these sites may be triggered by a larger pool of OCPs due to the disruption of IL-1 inhibitors.

## 1. Introduction

Osteoclasts are multinucleated bone-resorbing cells that are essential for the homeostasis of bone. Shifting the balance in bone turnover favoring osteoclasts may result in excessive bone destruction in diseases like rheumatoid arthritis, osteoporosis, and periodontitis as reviewed for these diseases in [1]. Various studies showed a correlation between rheumatoid arthritis and the development of periodontitis. It has been reported that patients with active rheumatoid arthritis have a significantly increased chance of developing periodontitis when compared with healthy controls and that patients with periodontal disease have a higher incidence of rheumatoid arthritis than patients without periodontitis [2,3]. Both diseases are associated with inflammation and high levels of proinflammatory cytokines, such as interleukins 1 α and β (IL-1α, β), interleukin 6 (IL-6), and tumor necrosis factor α (TNF-α). Particularly, IL-1β has been shown to strongly stimulate osteoclastogenesis and bone resorption [4,5,6].

IL-1β can bind to two receptors, type I IL-1 receptor (IL-1RI) and type II IL-1 receptor (IL-1RII). The endogenous inhibitor IL-1 receptor antagonist (IL-1RA) competitively blocks the binding of IL-1β or IL-1α to IL-1RI, thereby regulating its signaling [7]. Another inhibitory mechanism is mediated by IL-1RII, which exists both as a membrane-bound and soluble receptor. In contrast to IL-1RI, IL-1RII lacks the Toll/IL-1R domain at the cytoplasmic terminus and is therefore incapable of signaling [8]. IL-1β’s effects on bone destruction are counteracted by IL-1RA via a negative regulation of osteoclastogenesis as well as bone resorption [9].

Mice lacking the gene encoding for IL-1RA (*Il1rn*) are commonly used as a model of enhanced IL-1 signaling based on the finding that these mice show higher levels of various proinflammatory cytokines, including IL-1β, IL-6, and TNF-α [10]. These cytokines can stimulate bone metabolism by activating osteoclasts; therefore, *Il1rn-*deficient (*Il1rn^−/−^*) mice represent an ideal model to study enhanced IL-1 signaling on bone destruction [10]. *Il1rn^−/−^* mice have been shown to develop bone erosion at various skeletal sites, particularly the ankle joint and the jaw [10,11]. Upon aging, *Il1rn^−/−^* mice spontaneously develop inflammation in the ankle joint, showing synovial and peri-articular inflammation, cell infiltration, and articular erosions [10,12,13,14]. In contrast, spine and knee joints are less susceptible to developing local inflammation in the *Il1rn^−/−^* mouse model [11,15]. Previous studies showed that various mononuclear cell fractions isolated from mouse bone marrow as well as from human peripheral blood differ in their capacity to form osteoclasts and their response to proinflammatory cytokines [6,16,17,18], suggesting that dysregulation of IL-1 signaling can have diverse effects depending on the skeletal sites and their susceptibility to developing inflammation.

A number of studies described that osteoclasts isolated from different bones or differentiated from the bone marrow cells of various skeletal sites are not always identical [19,20,21,22]. The bone marrow of each skeletal site contains different subsets of osteoclast precursors [22]. Based on their surface markers, these subsets are recognized as early blasts (CD31^hi^ Ly-6C^−^), myeloid blasts (CD31^+^ Ly-6C^+^), and monocytes (CD31^−^ Ly-6C^hi^) [23]. These three subsets have the capacity to differentiate into osteoclasts, but they respond differently to macrophage colony-stimulating factor (M-CSF) and receptor activator of nuclear factor kappa-Β ligand (RANKL) [17] and to IL-1β [6] with respect to proliferation, multinucleation, life span, and bone resorption. In a previous study, it was shown that IL-1β stimulated the proliferation of early blasts. Although it induced multinucleation of all subsets, this effect was most pronounced in myeloid blast cultures [6]. Therefore, we hypothesized that a lack of IL1-RA increases the number of osteoclast precursors differently at various skeletal sites, which will influence the osteoclastogenic potential of the bone marrows. In the present study, using *Il1rn^−/−^* mice, we aimed to elucidate the effects of the absence of this negative regulator of IL-1 on the percentage of the three osteoclast precursor types in the bone marrow of long bone, calvaria, vertebra, and jaw. Subsequently, the consequence of a skewed distribution of osteoclast precursors in the skeletal bone marrows on osteoclast formation and resorption activity was assessed.

## 2. Results

### 2.1. Absence of Il1rn Significantly Increases the Number of Osteoclast Precursors, Particularly Monocytes, in the Long Bone and Jaw Marrow

**Figure 1:**
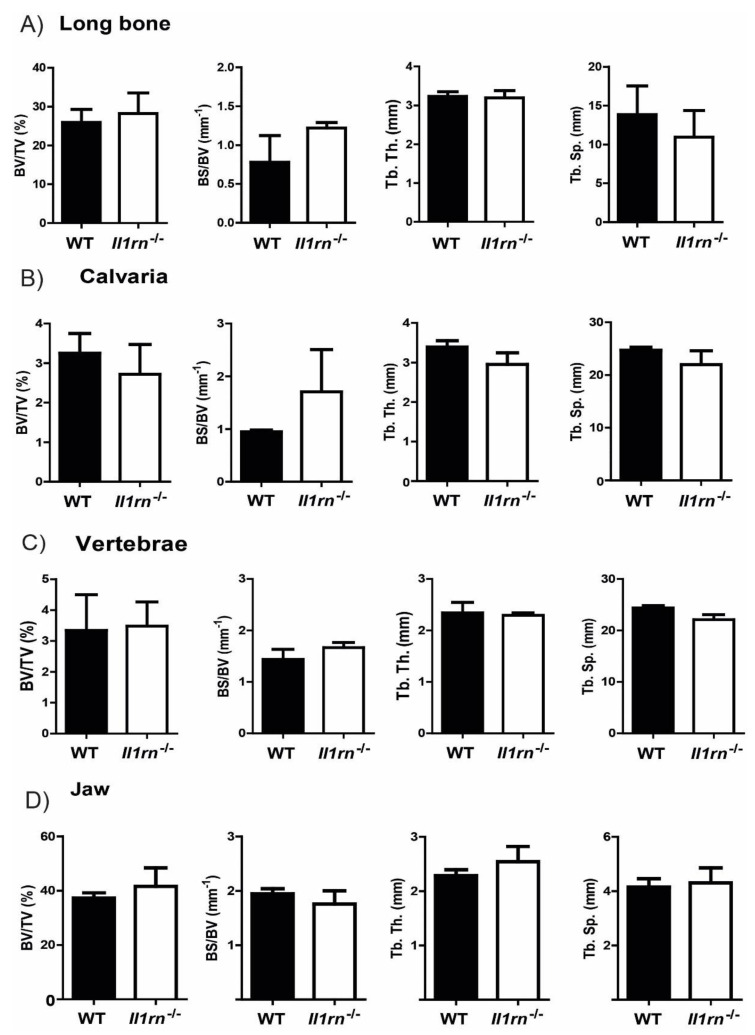
MicroCT analysis of WT and *Il1rn^−/−^* mice showed no differences in bone microstructure independently of the skeletal site. To assess possible bone destruction at the different skeletal sites, various 3-D microstructural bone parameters, including bone volume fraction (BV/TV), specific bone surface (BS/BV), trabecular thickness (Tb.Th.), and trabecular separation (Tb.Sp.), were measured in all specimens from *Il1rn^−/−^* mice and wild-type (WT) controls (**A**–**D**) (*n* = 3 mice/group). These parameters were comparable between the two mouse strains irrespective of the bone site, suggesting that these *Il1rn^−/−^* mice did not develop structural pathology yet.

We used *Il1rn*^−/−^ mice that had developed no detectable inflammation as determined by macroscopic scores (data not shown) of the ankle joints. Histological assessment of all four skeletal locations (long bone, calvaria, vertebrae, and jaw) did not show differences in the cellular composition as well as signs of bone erosion (Figure S1). In line with this, micro computed tomography (µCT) scans showed a similar bone volume fraction (BV/TV) ratio in the trabecular region in the various skeletal sites of WT and *Il1rn^−/−^* mice. Furthermore, the specific bone surface (BS/BV), trabecular thickness (Tb.Th.), and trabecular separation (Tb.Sp.), as other relevant parameters for the analysis of trabecular bone, were similar between WT and *Il1rn^−/−^* specimens (Figure 1A–D), suggesting that enhanced bone loss had not taken place yet.

**Figure 2:**
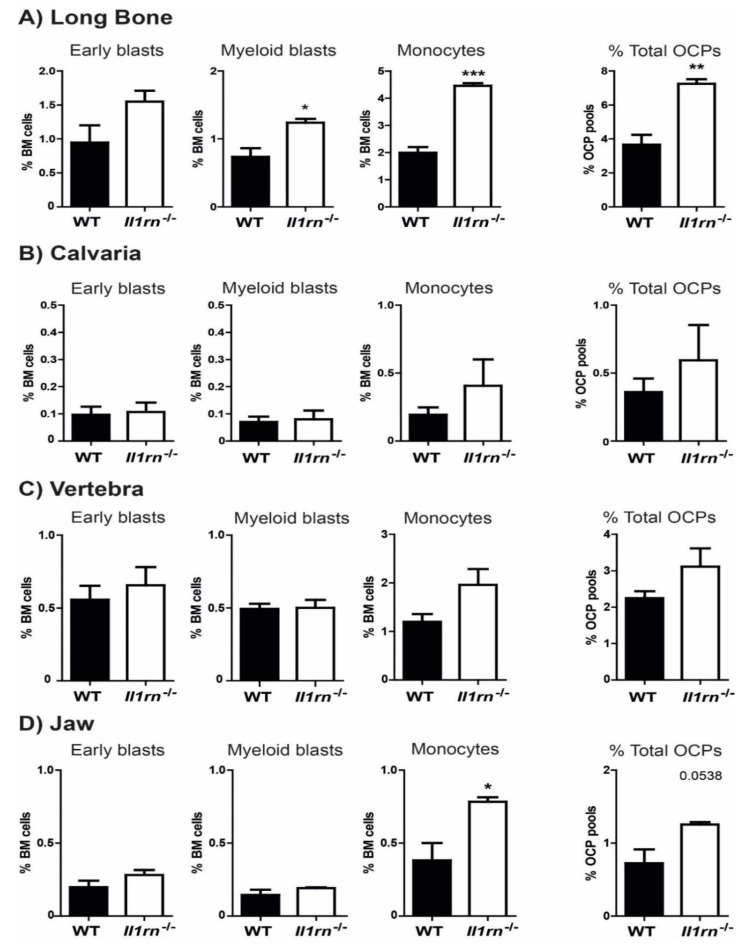
Absence of IL-1RA increases the percentage of myeloid blasts in long bone, and the percentage of monocytes in long bone and jaw. Percentage of early blasts, myeloid blasts, and monocytes from long bone (**A**), calvaria (**B**), vertebra (**C**), and jaw (**D**) were quantified and compared between wild-type (WT) (black bars) and *Il1rn^−/−^* (white bars) mice (*n* = 6, *p < 0.05 **p < 0.01, ***p < 0.001). Whereas the calvaria and vertebral bone marrow (BM) of *Il1rn^−/−^* mice showed no significant differences in the percentage of various osteoclast precursor subsets, we found a significant increase of both the myeloid blasts and monocyte subsets in the BM of long bone. Similarly, the monocyte subset of OCPs was also significantly increased in the jaw BM of *Il1rn^−/−^* mice as compared to WT controls.

After we established the absence of structural bone pathology in these *Il1rn*^−/−^ mice that had not developed visible inflammation, we explored using flow cytometry whether the absence of IL-1RA affected the different osteoclast precursor subsets in bone marrow (early blasts, myeloid blasts, and monocytes) (Figure S2). Interestingly, we observed remarkable differences among the percentage of total osteoclast precursors in bone marrow derived from different skeletal sites (Figure 2A–D). An increase of the three subsets was observed in long bones of *Il1rn^−/−^* mice, where the total percentage of osteoclast precursors was significantly increased (from around 4% in WT to 7% in *Il1rn^−/−^* mice). In particular, the percentage of monocytes was increased more than 2-fold (from 2.0% in WT to 4.4% in *Il1rn^−/−^* mice) (Figure 2A), as well as that of myeloid blasts (increase from 0.7% in WT to 1.2% in *Il1rn^−/−^* mice). Additionally, the marrow of the jaw showed a 2-fold increase of the monocyte subset (from 0.4% in WT to 0.8% in *Il1rn^−/−^* mice) (Figure 2D). In contrast to the long bone and jaw, no differences in the percentual contribution were found in any of the osteoclast precursor pools between the calvariae (Figure 2B) and vertebrae (Figure 2C) of WT and *Il1rn^−/−^* mice.

### 2.2. Enhanced Osteoclast Formation and Multinucleation by Bone Marrow Cells Derived from the Long Bone, Calvaria, and Jaw of Il1rn^−/−^ Mice

**Figure 3:**
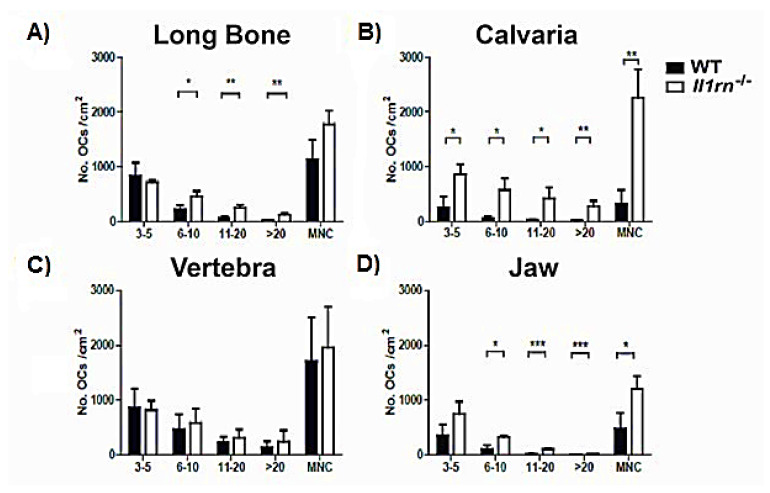
Increased number of osteoclasts generated from long bone, calvaria, and jaw but not vertebra marrow cells of *Il1rn^−/−^* mice. Bone marrow cells from long bone, calvaria, vertebra, and jaw were cultured with 30 ng/mL M-CSF and 20 ng/mL RANKL on bone slices for 6 days. Osteoclasts were stained for tartrate resistant acid phosphatase (TRAcP), counted, and categorized as 3–5 nuclei, 6–10 nuclei, 11–20 nuclei, and >20 nuclei. The number of osteoclasts (≥3 nuclei) was counted for long bone (**A**), calvaria (**B**), vertebra (**C**), and jaw (**D**) and compared between wild-type (WT) and *Il1rn^−/−^* mice. The total number of osteoclasts is shown as multinucleated cells (MNCs). Osteoclastogenesis was significantly higher in long bone, calvaria, and jaw of *Il1rn^−/−^* osteoclast precursors (OCPs) compared to WT OCPs. In contrast, WT and *Il1rn^−/−^* OCPs isolated from vertebrae formed a comparable number of osteoclasts (*n* = 6 mice/group, *p < 0.05, **p < 0.01, ***p < 0.001).

We next investigated whether the loss of IL-1RA altered the potential to induce osteoclast differentiation in vitro on bone slices. Bone marrow cells from the four skeletal sites containing all subsets of osteoclast precursors were seeded on bone slices and cultured for 6 days in the presence of M-CSF and RANKL. Osteoclast precursors present in the bone marrow obtained from WT and *Il1rn*^−/−^ specimens were all able to differentiate into multinucleated and TRAcP^+^ osteoclasts (Figure 3A). Osteoclasts were classified depending on the number of their nuclei (3–5; 6–10; 11–20; and >20), a parameter that positively correlates with osteoclast size.

*Il1rn^−/−^* bone marrow cells from long bone, jaw, and calvaria showed significantly increased osteoclastogenesis as compared to their WT controls (Figure 3A,B,D). Particularly, *Il1rn*^−/−^ cells from the long bone and jaw gave rise to a higher number (2- and 3-fold higher, respectively) of intermediate and large osteoclasts (>5 nuclei and >10 nuclei, respectively). The enhanced osteoclastogenesis in *Il1rn^−/−^* cells from calvaria (4 times higher as compared to WT control cells) was due to an increase of intermediate and large osteoclasts (>5 nuclei and >10 nuclei, respectively) but also of small osteoclasts (3–5 nuclei). In contrast, a comparable number of osteoclasts from the vertebral bone marrow of WT and *Il1rn^−/−^* mice was observed.

**Figure 4:**
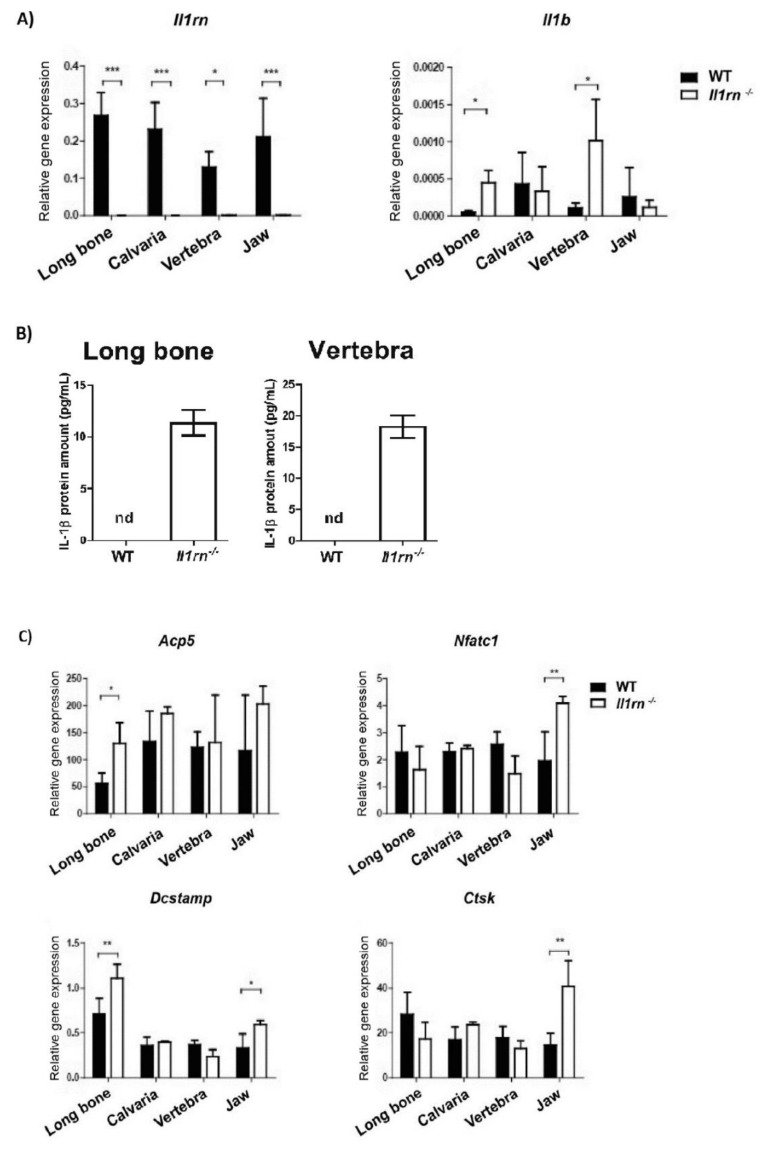
Absence of IL1-RA induces the expression of osteoclast markers particularly in long bone and jaw-derived osteoclasts. Gene expression was measured in wild-type (WT) and *Il1rn^−/−^* osteoclasts derived from long bone, calvaria, vertebra, and jaw bone marrow (BM) that were cultured on bone slices for 6 days. (**A**) Cultures of *Il1rn^−/−^* osteoclasts from all skeletal sites show no expression of *Il1rn.* The absence of *Il1rn* significantly increased the mRNA expression of *Il1b* in long bone and vertebra-derived osteoclasts (*n* = 6 mice/ group). (**B**) Protein levels of IL1-β were determined in the culture supernatants at day 3 of osteoclastogenesis. Whereas IL1-β production was below the detection limit in WT and *Il1rn^−/−^* osteoclasts from calvaria and jaw, *Il1rn^−/−^* osteoclasts derived from long bones and vertebrae showed increased IL1-β levels (*n* = 3 mice/group). nd = not detected. (**C**) Of note, the mRNA expression of various specific osteoclast markers was significantly increased exclusively in *Il1rn^−/−^* osteoclasts derived from long bone and jaw BM (*n* = 6 mice/group). Acid phosphatase 5 (*Acp5*); nuclear factor of activated T cells 1 (*Nfatc1*); dendritic cell-specific transmembrane protein (*Dcstamp*); cathepsin K (*Ctsk*). All values were normalized for the expression of Beta 2 microglobulin (*B2m*) as the reference gene. Relative expression is shown (* = *p* < 0.05, ** = *p* < 0.01, *** = *p* < 0.001 compared to WT controls).

qRT-PCR analysis revealed that all cultures of WT osteoclasts expressed *Il1rn*, whereas, as expected, no expression was detected in the *Il1rn^−/−^* osteoclasts obtained from any of the skeletal sites (Figure 4A). Osteoclasts generated from *Il1rn^−/−^* bone marrow expressed *Il1b*, suggesting that IL-1 secreted by these cells may contribute to enhanced IL-1 signaling after isolation (Figure 4A). In line with this, IL-1β production was found exclusively in the culture supernatants of *Il1rn^−/−^* osteoclast derived from long bone and vertebra at day 3 of osteoclastogenesis (Figure 4B). However, IL-1β was not detectable in any sample at a later time-point during osteoclastogenesis (day 6), indicating that the priming effect is lost when the precursors are taken out of their bone microenvironment. These data suggest that not only the absence of IL-1RA increases IL-1β production, hence IL-1 signaling, but also that the latter is differently regulated at different skeletal sites. IL-1 expression was induced by priming in endochondral bone environment (long bones and vertebra) and not the endesmal bone environment (calvaria and jaw). Of interest, and in agreement with an increased number of osteoclasts being formed, a lack of IL-1RA resulted in a significant increase of various specific osteoclast markers (*Acp5, Nfatc1, Dcstamp, Ctsk*) in *Il1rn^−/−^* osteoclasts derived from long bone and jaw marrow but not from calvaria and vertebra (Figure 4C).

### 2.3. Il1rn^−/−^ Osteoclasts Show Increased Mineral Dissolving Capacity on Calcium Phosphate-Coated Plates Independently of the Skeletal Site

**Figure 5:**
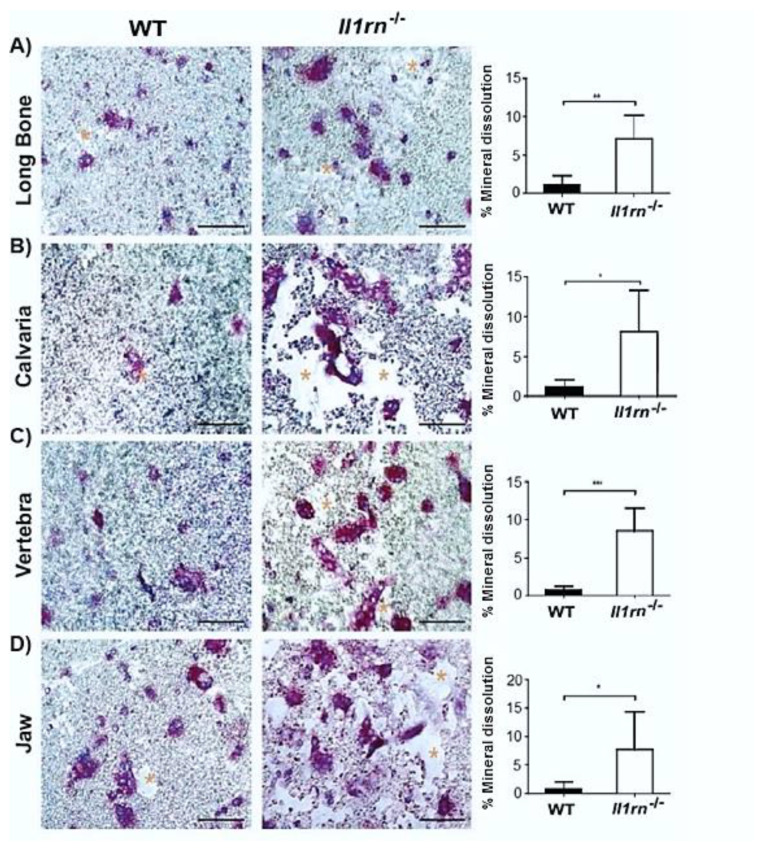
*Il1rn^−/−^* osteoclasts show an enhanced mineral dissolution independently of the skeletal site. Absence of IL-1RA increased the in vitro dissolution of the inorganic matrix of bone from all osteoclast precursors irrespective of the isolation site, suggesting that site-specific differences present in vivo can be outweighed in vitro. (**A**–**D**) Microphotographs of hydroxyapatite-coated plates of which part is dissolved by osteoclasts derived from various skeletal sites in both wild-type (WT) and *Il1rn^−/−^* mice. Bone marrow (BM) cells from long bone, calvaria, vertebra, and jaw were cultured with 30 ng/mL M-CSF and 20 ng/mL RANKL on hydroxyapatite-coated plates for 8 days. Osteoclasts were stained by TRAcP (in purple) and nuclei were stained by 4’,6-Diamidino-2-Phenylindole (DAPI) (blue). The dissolved area was labeled by asterisks. Scale bar = 100 µm. The dissolved area was quantified and compared between WT and *Il1rn^−/−^* mice for long bone (**A**), calvaria (**B**), vertebra (**C**) and jaw (**D**) (*n* = 6, *p < 0.05, **p < 0.01, ***p < 0.001).

We seeded the bone marrow cells from the four skeletal sites on calcium phosphate-coated plates to determine whether the cells that were cultured with M-CSF and RANKL were able to dissolve mineral. The absence of *Il1rn* resulted in an increased dissolvement of the coating by cells from all skeletal sites, as indicated by the larger areas devoid of mineral as compared to WT control osteoclasts (Figure 5A–D). Quantification of the dissolved area showed a sharp increase of this parameter (~8-fold higher). Together, these findings suggest that a lack of IL-1RA significantly increases the lysis of mineral independently of the skeletal site they were isolated from.

## 3. Discussion

The present study showed that in the absence of IL-1RA, priming of osteoclast precursors in bone marrow was to some extent bone site specific. In particular, the relative contribution of monocytes was increased in the bone marrow of the long bone and jaw when compared to calvaria and vertebra. Further, we showed that the absence of IL-1RA significantly increased the in vitro formation of osteoclasts and mineral dissolution irrespective of the site of isolation.

*Il1rn^−/−^* mice are commonly used as a model for studying bone loss during arthritis; however, little is known about how osteoclastogenesis and resorptive activity is regulated at different skeletal sites prior to the onset of inflammation and bone destruction. In this study, we used a subset of mice that showed no signs of inflammation or bone destruction in any of the investigated skeletal sites, as shown by both microCT bone parameters and qualitative assessment of histology.

It has been demonstrated that *Il1rn^−/−^* mice develop inflammation at some preferential skeletal sites like the ankle and jaw [10,11,24]. Ankle joints showed marked invasion of inflammatory cells and osteoclast activation resulting in bone destruction [10]. Similarly, *Il1rn^−/−^* mice infected with periodontopathogenic bacterium *A. actinomycetemcomitans* showed a high number of inflammatory cells and an increased osteoclast number adjacent to the alveolar bone as well as loss of epithelial attachment, a central feature of periodontitis [24]. Further, the absence of IL-1RA caused inflammation in the vertebrae of ageing mice, resulting in a loss of proteoglycan and increased expression of matrix-degrading enzymes. However, the bone abnormalities observed in *Il1rn^−/−^* mice at this skeletal site are mostly due to increased trabecular bone [15] and little is known about the effects of the absence of IL-1RA on vertebral osteoclasts. Interestingly, in ankylosing spondylitis, an inflammatory disease that particularly affects the vertebrae, both reduced and increased bone formation is observed, suggesting a dual effect of inflammation on bone metabolism at this particular bone site [25].

It is known that proinflammatory cytokines, such as TNF-α, can expand the number of osteoclast precursors in inflammatory models of rheumatoid arthritis [26,27]. However, little is known about the effects of IL-1 on the expansion of various osteoclast precursors in vivo. In this study, a lack of IL-1 dampener IL-1RA resulted in a higher percentage of in particular monocytes in the bone marrow of long bone and jaw. Monocytes are the most recruited osteoclast precursors during inflammation [28] and osteoclasts derived from monocytes have a longer lifespan as compared to osteoclasts formed from early blasts or myeloid blasts [6]. In another study, using a TNF-α transgenic mouse that also show increased production of IL-1, the number of osteoclast precursors (CD11b^high^) was increased [29].

Monocytes and polymorphonuclear cells are potent producers of proinflammatory cytokines that can boost bone destruction during inflammatory diseases characterized by excessive bone destruction [30]. In a previous study, it was shown that among the various osteoclast precursor subsets, particularly myeloid blasts give rise to large osteoclasts when stimulated by IL-1β [6]. Therefore, the increased percentage of myeloid blasts observed in long bone marrow, and monocytes in both long bone and jaw marrow in conjunction with the lack of IL-1RA may lead to larger osteoclasts and probably increased resorption when local inflammation is present. Noteworthy, long bone marrow contained a much higher percentage of osteoclast precursors when compared to the other bone sites. This in conjunction with their enhanced IL-1β production may boost bone destruction particularly at this bone site. Moreover, it has to be noted that an increased production of IL-1β was found exclusively in osteoclast precursors from long bone and vertebra. This hints that bones undergoing endochondral ossification (i.e., long bones and vertebrae) respond differently as compared to those formed via intramembranous ossification (i.e., calvaria and jaw). These findings suggest that IL-1 is not only able to activate the precursors via triggering of the osteoclastogenic signalling pathway [5] but that it may also site specifically steer the composition of osteoclast precursor pools towards the monocyte subset. However, as IL-1β has cell-type-specific effects, thus inducing neutrophilia, leukocytosis, and thrombocytosis in vivo [31,32], it is conceivable that the lack of IL-1RA may enhance IL-1 signaling, which stimulated the proliferation of specific osteoclast precursor subsets.

Noteworthy, the lack of IL-1RA resulted in a significant increase of various specific osteoclast markers exclusively in *Il1rn^−/−^* osteoclasts derived from the long bone and jaw marrow. Interestingly, increased bone loss due to enhanced osteoclast formation has been noticed in *Il1rn^−/−^* mice after the induction of periodontitis [24,33] as well as in rheumatoid arthritis [10,11]. Previous studies have shown a strong correlation between active rheumatoid arthritis, characterized by high-grade inflammation and joint destruction, and progression of periodontal disease [34]. Protein citrullination [35], the composition of the subgingival microbiome [36], and systemic inflammation [37] are factors that have been suggested to link rheumatoid arthritis to periodontitis. The production of proinflammatory cytokines, such as TNF-α, IL-1β, and IL-6, in both synovial and periodontal tissues triggers similar immune responses, which drive periodontal inflammation, ultimately leading to joint destruction and tooth bone loss, respectively [38]. Together, our findings indicate that the lack of IL-1RA in vivo site-specifically increases the monocyte subset of osteoclast precursors and supports the hypothesis that local differences in osteoclast precursors and their response to cytokines can exacerbate bone destruction in the long bone and jaw, thus representing an additional mechanistic link between rheumatoid arthritis and periodontitis.

In contrast to the long bone and jaw, enhanced IL-1 signaling did not significantly alter the composition and percentage of osteoclast precursors in the vertebral and calvaria bone marrow. As we found that the deletion of *Il1rn* equally boosted the mineral dissolution by in vitro differentiated osteoclasts for all skeletal locations, this suggests that enhanced IL-1 signaling makes osteoclasts more active. Enhanced bone destruction in the vertebrae is mainly observed in age-related and metabolic disorders, such as osteoporosis [39]. Of interest, although a loss of IL1-RA did not affect the percentage of osteoclast precursors in vertebral bone marrow, *Il1rn^−/−^* osteoclasts derived from this skeletal site showed a significant increase of both *Il1b* mRNA and protein levels when compared to their WT controls. As systemic inflammation is associated with an increased risk of developing osteoporosis in older subjects, it may be that enhanced IL-1 signaling increases osteoclast activity in the vertebrae only in combination with ageing, probably explaining the lack of structural pathology observed in the vertebrae of *Il1rn^−/−^* mice. Although the skull microenvironment strongly reacts to inflammatory stimuli [40,41], bone destruction at this site predominantly occurs in rare skeletal disorders like Paget’s disease [42], where the disruption of bone metabolism rather than inflammation is the central feature, or after an injury as part of the healing process [43]. In the present study, we showed that all osteoclast sizes are increasingly formed from calvarial bone marrow (Figure 3), independently of changes in the composition (Figure 2) or changes in gene expression (Figure 4). This could suggest that the absence of IL-1RA together with the use of optimal concentrations of M-CSF and RANKL used to induce osteoclastogenesis make the osteoclast precursors more active, overruling the local differences present in vivo. Therefore, additional in vitro studies using suboptimal concentrations of M-CSF and RANKL would help to determine the osteoclastogenic potential at the various skeletal sites. It was previously shown that osteoclasts from long bone and calvaria use different proteolytic enzymes to digest the bone matrix [20,44], and they make use of different ion transporters to modulate the intracellular pH [45]. Furthermore, a previous study showed that long bone marrow has a higher osteoclastogenic potential as compared to cells from the jaw marrow [22]. This may be due to bone-specific differences in the hemopoietic niche present in the bone marrow that differently stimulate osteoclast precursors. We propose that osteoclast precursors present at different skeletal locations may give rise to phenotypically different osteoclasts, probably explaining the lower susceptibility of *Il1rn^−/−^* mice to develop erosions in the spine [15] and the lack of an enhanced in vivo resorption of the calvarial bone in the absence of an inflammatory trigger [33].

We hypothesize that different skeletal locations might modulate osteoclastogenesis differently in case of possible excessive IL-1 signaling, such as in mice deficient for IL-1RA. This study provides new insights into the osteoclast differentiation of bone marrow cells from different skeletal sites induced by the absence of IL-1RA and further highlights the diversity of IL-1 signaling regulation at different skeletal sites. Collectively, our data suggest that possible sustained IL-1 signaling by the deletion of *Il1rn* elicited a skeletal site-dependent response in the bone marrow composition of the long bone and jaw, and this can represent an additional link between rheumatoid arthritis and periodontal pathology.

## 4. Materials and Methods

### 4.1. Il1rn^−/−^ Mice

*Il1rn^−/−^* mice on Balb/c background were kindly supplied by Dr. M. Nicklin (University of Sheffield, Sheffield, UK) and generated as previously described [46]. The mice were housed in conventional filter-top cages, and food and water were provided ad libitum. Age-matched Balb/c mice were used as controls for all experiments. All animal studies were approved by the institutional animal ethics committees from the Vrije Universiteit (VU), Amsterdam, The Netherlands (ACTA- DEC 2014-1) and the Radboud university medical center (Radboudumc), Nijmegen, The Netherlands (RU-DEC 2014-116).

### 4.2. Histology

Tissue samples from wild-type (WT) and *Il1rn^−/−^* mice were fixed in 4% paraformaldehyde, decalcified in ethylenediaminetetraacetic acid (EDTA) and subsequently embedded in paraffin wax. Longitudinal sections (7 µm thick) of long bone, calvaria, vertebrae, and lower jaw bone were stained with hematoxylin and eosin (H&E) for qualitative assessment of the presence of immune cells as a parameter for inflammation.

### 4.3. Microcomputed Tomography (µCT)

Femurs, calvariae, vertebrae, and jaws were dissected from WT or *Il1rn^−/−^* mice and fixed with 4% paraformaldehyde for 48 h, then stored in 70% ethanol at 4 °C. Scans were obtained with a SCANCO Medical machine and ex vivo µCT analyses were performed with Skyscan 1172 Software (Bruker, Germany). The region of interest (ROI) was set using a manually determined global threshold. Three-dimensional microstructural bone properties, including the bone volume fraction (BV/TV), specific bone surface (BS/BV), trabecular thickness (Tb.Th.), and trabecular separation (Tb.Sp.), were calculated according to the manufacturer’s software.

### 4.4. Bone Marrow Isolation

*Il1rn^−/−^* and WT male mice were sacrificed between 14 and 16 weeks of age and only mice that did not show any signs of macroscopic inflammation at the ankle were further analyzed. Mice were killed with a peritoneal injection of a lethal dose of sodium pentobarbitone (Euthesate, 0.1 mL sodium pentobarbital per mouse; Sanofi Santé Animale Benelux, Maassluis, The Netherlands). Bone marrow cells were isolated from four skeletal sites: Long bone (both femur and tibia), calvaria, vertebra, and lower jaw bone. Soft tissue was removed from the bones using scissors and a scalpel knife. All bones were mashed in a mortar with 5 mL of α-MEM (Gibco; Thermo Fisher Scientific, Paisley, Scotland) supplemented with 5% fetal calf serum (HyClone; GE Healthcare Life Sciences, Logan, UT, USA) and 1% penicillin-streptomycin-fungizone (Sigma-Aldrich, St. Louis, MO, USA). The released cells were aspirated through a 21-gauge needle and the suspended bone marrow cells were filtered through a 40-μm filter. The number of cells were counted using a MUSE^TM^ cell analyzer (Merck, Darmstadt, Germany).

### 4.5. Immunofluorescence Labeling and Flow Cytometric Analysis

First, bone marrow cells were incubated with Fc-blocking antibody (BD Pharmingen anti-mouse CD16/CD32, clone 2.4G2; BD Biosciences, San Jose, CA, USA), followed by staining with a byotinilated anti-CD31 antibody (AbD Serotec, Kidlington, UK) diluted in FACS buffer (1% albumin from BSA [Sigma-Aldrich]). After 30 min, samples were incubated with an Alexa 488-labeled anti-Ly-6C antibody (AbD Serotec, Kidlington, UK) diluted in FACS buffer containing streptavidin PE (Becton Dickinson, San Jose, CA, USA). Cell viability was assessed by using SYTOX blue viability dye and the percentage of early blasts (CD31^hi^ Ly-6C^−^), myeloid blasts (CD31^+^ Ly-6C^+^), and monocytes (CD31^−^ Ly-6C^hi^) were measured by Gallios Flow Cytometer. Debris, which typically has both low forward scatter and low sideward scatter, is excluded from the gating strategy. Analyses were performed using the Kaluza Analysis software 1.3 (Beckman Coulter, Brea, CA, USA).

### 4.6. Cell Culture

Bone marrow cells isolated from the four skeletal sites were seeded at a density of 10^5^ cells/well in 96-well plates (Cellstar; Greiner Bio-One, Monroe, NC, USA) and cultured in α-MEM medium containing 30 ng/mL of macrophage-colony stimulating factor (M-CSF) (R&D Systems, Minneapolis, MN, USA) and 20 ng/mL of receptor activator nuclear factor kappa-B ligand (RANKL) (RANKL-TEC, R&D Systems). Culture media were refreshed after 3 days, and cultures were stopped after 6 days and either fixed in 4% formaldehyde for tartrate resistant acid phosphatase (TRAcP) staining, or lysed in RNA lysis buffer (Qiagen, Hilden, Germany) to determine gene expression using quantitative real-time polymerase chain reaction (qRT-PCR).

### 4.7. Analysis of Tartrate-Resistant Acid Phosphatase Positive Cells

Cells cultured on bone slices were fixed and stained for TRAcP using a commercially available leucocyte acid phosphatase kit (Sigma-Aldrich). The staining procedure was performed following the manufacturer’s instructions and has been described previously [6]. Nuclei were counterstained by 4′6-diamidino-2-phenylindole (DAPI), and the number of TRAcP-positive (TRAcP^+^) cells with three or more nuclei was assessed and categorized into four groups: 3–5, 6–10, 11–20, and >20 nuclei. The number of TRAcP^+^ cells in each category was counted using a combination of light and fluorescence microscopy (Leica DFC320; Leica Microsystems, Wetzlar, Germany). Results were expressed as the number of osteoclasts per cm^2^.

### 4.8. Calcium Phosphate Coating and Analysis of Areas of Lysis

Calcium phosphate-coated 96-well plates were prepared as previously described [47]. This coating is often used to study the mineral dissolving capacity of cells, among them osteoclast-like cells/cells that were differentiated with M-CSF and RANKL [48]. The coated 96-well plates were sterilized by UV light exposure. The cultures were fixed in 4% paraformaldehyde after 8 days and the resorbed area in relation to the total coating area was visualized by light microscopy (Leica DFC320), and quantified using Image Pro Plus (Media Cybernetics, Silver Spring, MD, USA).

### 4.9. Quantitative RT-PCR

Messenger RNA (mRNA) expression of *Il1rn* and *Il1b*, the encoding genes for IL-1RA and IL-1β, respectively, was measured by RT-PCR as previously described [6]. In addition, the mRNA expression of the tartrate resistant acid phosphatase 5 (*Acp5*), cathepsin K (*Ctsk*), nuclear factor of activated T cells 1 (*Nfatc1*), and dendritic cell-specific transmembrane protein (*Dcstamp*) as osteoclast markers was also determined. Samples were normalized for the expression of the housekeeping gene *B2m* encoding for Beta 2 Microglobulin. The primer sequences used were the following: for *B2m*, FW:TGCTATCCAGAAAACCCCTCAA, RV: GCGGGTGGAACTGTGTTACG; for *Il1rn*, FW: TGTGCCAAGTCTGGAGATGATATC, RV: TTGTTCTTGCTCAGATCAGTGATG; for *Il1b*FW: GGACCCATATGAGCTGAAAGCT, RV: TGTCGTTGCTTGGTTCTCCTT; for *Acp5* FW: GACAAGAGGTTCCAGGAGACC, RV: GGGCTGGGGAAGTTCCAG; for *Ctsk* FW: ACAGCAGGATGTGGGTGTTCA, RV: GCCGAGAGATTTCATCCACCT; for *Nfatc1* FW: GAGTTGTGCAATGGCAATTCTG, RV: TGGTAGCATCCATCATTTCTTTGT; for *Dcstamp* FW: TGTATCGGCTCATCTCCTCCAT, RV: GACTCCTTGGGTTCCTTGCTT. The relative expression of each gene was calculated as 2^−ΔCt^, ΔCt  =  (Ct _gene of interest-_ Ct _housekeeping gene_) and the results were shown as the relative expression.

### 4.10. Luminex

Protein levels of IL-1β were measured in culture supernatants using Luminex multianalyte technology on the Bio-Plex 100 system and multiplex cytokine kit (Bio-Rad, Hercules, CA, USA), the sensitivity of which was < 1 pg/mL.

### 4.11. Statistical Analysis

Differences between WT and *Il1rn^−/−^* mice were tested by Student’s *t*-test or non-parametric Mann–Whitney U test when a non-Gaussian distribution was assumed (*n* = 3) using GraphPad Prism (version 6.00; GraphPad Software, LaJolla, CA). Data were expressed as mean ± SD. *p* < 0.05 was considered as a significant difference.

## Supplementary Materials

The following are available online at https://www.mdpi.com/1422-0067/21/11/3774/s1.
