# Supplementary material for: Increase in the Number of Bone Marrow Osteoclast Precursors at Different Skeletal Sites, Particularly in Long Bone and Jaw Marrow in Mice Lacking IL-1RA
Source: Int J Mol Sci. 2020 May 27;21(11):3774. doi: 10.3390/ijms21113774 (PMC7312984; doi:10.3390/ijms21113774)
Supplement: Supplementary file 1 [file ijms-21-03774-s001.pdf]

Supplementary figures

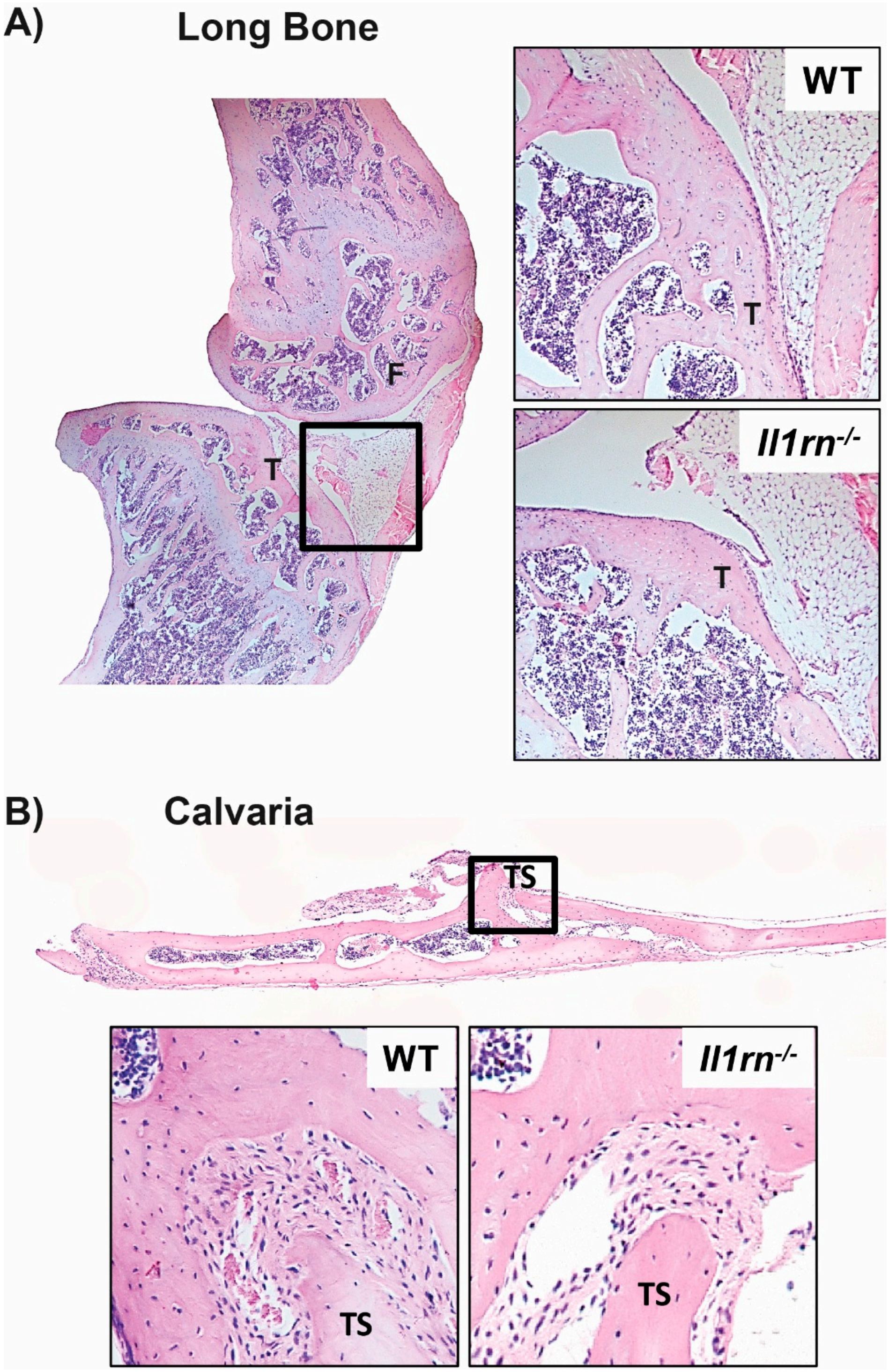

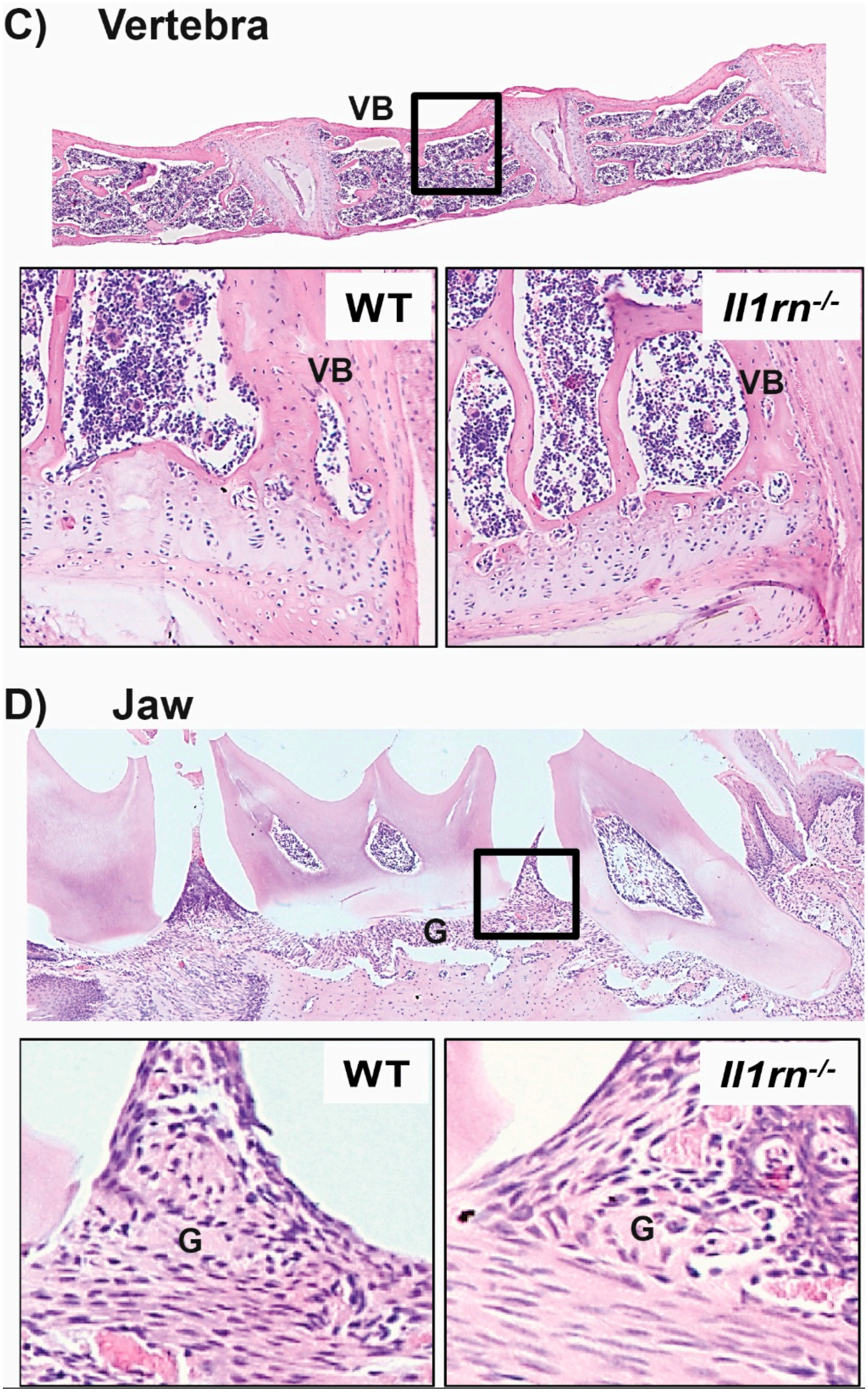

Figure S1. Comparable presence of immune cells in WT and *Il1rn*$^{-/-}$ specimens. Representative microphotographs of long bone (**A**), calvaria (**B**), vertebra (**C**), and jaw (**D**) in WT and *Il1rn*$^{-/-}$ mice (*n*=3 mice/group). Qualitative assessment of the presence of immune cells as parameter for tissue inflammation was performed in mice that showed no signs of macroscopic inflammation. In long bone the presence of immune cells was assessed at the articulating surface of the joint (**A**) and at the transsuture in the calvaria bones (**B**). In the vertebrae, the presence of immune cells was assessed in the distal part of the vertebral body in close proximity to the cartilage tissue (**C**). In the jaw the presence of cells was determined in the gum (**D**). In all skeletal sites no differences in the presence of possible immune cells within the tissue were found. Original magnification 100x. **F**= femur; **T**= tibia; **TS**= transsuture; **VB**= vertebral body; **G**= gum.

A)

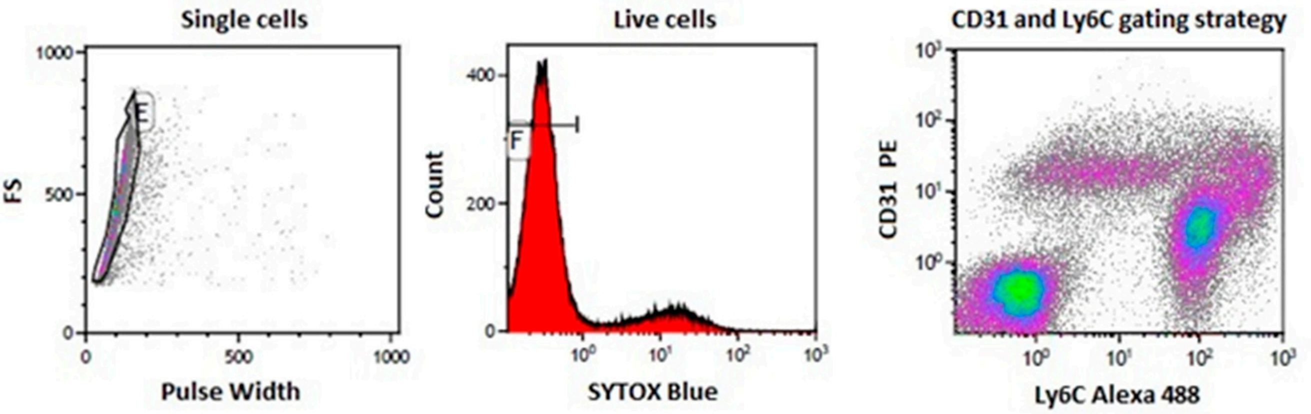

Single cells
FS
Pulse Width
E
Live cells
Count
SYTOX Blue
F
CD31 and Ly6C gating strategy
CD31 PE
Ly6C Alexa 488

B)

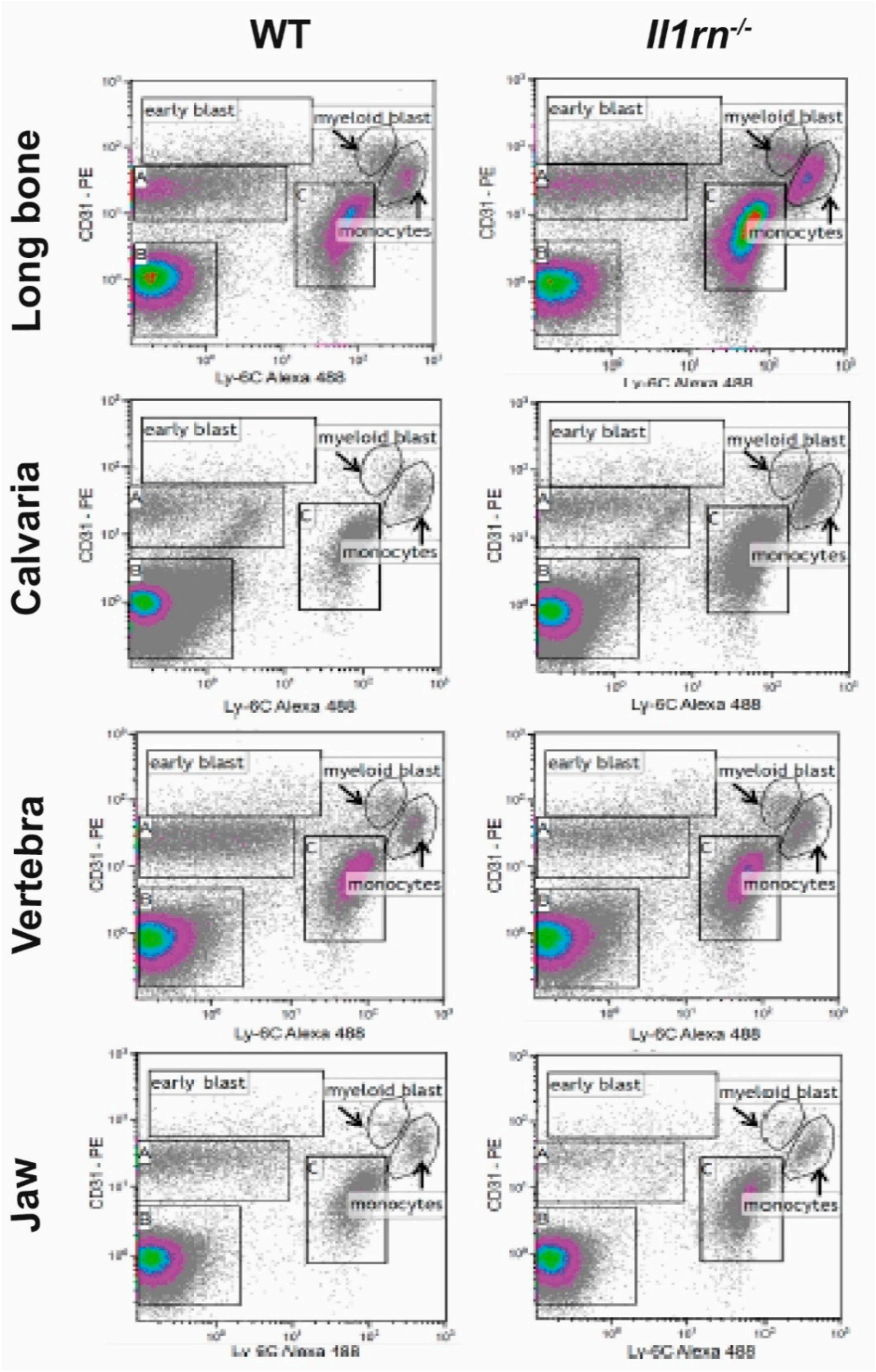

Figure S2. Gating strategy for flowcytometric analysis and identification of different subsets of osteoclast precursors in bone marrow from long bone, calvaria, vertebra and jaw in WT and *Il1rn*$^{-/-}$mice. **A)** First, single cells were selected. Subsequently, viable cells were discriminated using SYTOX Blue viability dye, where the live cells are represented by the negative fraction. Live cells were then gated for their expression of CD31 and Ly6C. **B)** Cells were labeled with anti-CD31 and -Ly-6C and osteoclast precursor subsets were gated as: early blasts (CD31^{hi} Ly-6C^{-}), myeloid blasts (CD31^{+} Ly-6C^{+}), and monocytes (CD31^{-} Ly-6C^{hi}) as encircled in each profile. Other populations mainly contained lymphocytes (Labeled A), erythroid blasts (Labeled B) and granulocytes (Labeled C).
